# Toxicity Screening of a *Gambierdiscus australes* Strain from the Western Mediterranean Sea and Identification of a Novel Maitotoxin Analogue

**DOI:** 10.3390/md19080460

**Published:** 2021-08-15

**Authors:** Pablo Estevez, David Castro, José Manuel Leão-Martins, Manoëlla Sibat, Angels Tudó, Robert Dickey, Jorge Diogene, Philipp Hess, Ana Gago-Martinez

**Affiliations:** 1 Biomedical Research Centre (CINBIO), Department of Analytical and Food Chemistry, University of Vigo, Campus Universitario de Vigo, 36310 Vigo, Spain; paestevez@uvigo.es (P.E.); dcastro@uvigo.es (D.C.); leao@uvigo.es (J.M.L.-M.); 2 Laboratoire Phycotoxines, Ifremer, Rue de l’Île d’Yeu, 44311 Nantes, France; manoella.sibat@ifremer.fr (M.S.); philipp.hess@ifremer.fr (P.H.); 3 Marine and Continental Waters Programme, Institut de Recerca i Tecnologies Agroalimentàries (IRTA), Ctra. Poble Nou, km. 5.5, 43540 Sant Carles de la Ràpita, Spain; angels.tudo@irta.cat (A.T.); Jorge.Diogene@irta.cat (J.D.); 4 Department of Marine Science, Marine Science Institute, University of Texas at Austin, Port Aransas, TX 78373, USA; robert.dickey@utexas.edu

**Keywords:** N2a, LC-MS/MS, LC-HRMS, maitotoxin, ciguatoxin, ciguatera poisoning

## Abstract

Dinoflagellate species of the genera *Gambierdiscus* and *Fukuyoa* are known to produce ciguatera poisoning-associated toxic compounds, such as ciguatoxins, or other toxins, such as maitotoxins. However, many species and strains remain poorly characterized in areas where they were recently identified, such as the western Mediterranean Sea. In previous studies carried out by our research group, a *G. australes* strain from the Balearic Islands (Mediterranean Sea) presenting MTX-like activity was characterized by LC-MS/MS and LC-HRMS detecting 44-methyl gambierone and gambieric acids C and D. However, MTX1, which is typically found in some *G. australes* strains from the Pacific Ocean, was not detected. Therefore, this study focuses on the identification of the compound responsible for the MTX-like toxicity in this strain. The *G. australes* strain was characterized not only using LC-MS instruments but also N2a-guided HPLC fractionation. Following this approach, several toxic compounds were identified in three fractions by LC-MS/MS and HRMS. A novel MTX analogue, named MTX5, was identified in the most toxic fraction, and 44-methyl gambierone and gambieric acids C and D contributed to the toxicity observed in other fractions of this strain. Thus, *G. australes* from the Mediterranean Sea produces MTX5 instead of MTX1 in contrast to some strains of the same species from the Pacific Ocean. No CTX precursors were detected, reinforcing the complexity of the identification of CTXs precursors in these regions.

## 1. Introduction

Species of dinoflagellates of the genera *Gambierdiscus* and *Fukuyoa* are known to produce toxic metabolites [1]. Ciguatoxins (CTXs) and maitotoxins (MTXs) are included among these toxic compounds, as are some other non-structurally-related cyclic polyethers, such as gambierol, gambieroxide, gambierone and gambieric acids [2,3,4,5,6]. 

CTXs are cyclic polyethers of around 1100 Da and considered the main compounds responsible for ciguatera poisoning (CP) due to their lipophilic nature and their accumulation in fish tissue [7]. Depending on their structural differences, CTXs are classified as P-CTXs, C-CTXs or I-CTXs [8,9,10]. The algal precursors of P-CTXs were identified in dinoflagellate cultures from the Pacific Ocean, being oxidized when accumulated in fish [11]. In contrast, CTX precursors were not identified in dinoflagellate cultures from the Indian or Atlantic oceans, including the Caribbean Sea, thereby limiting the detection of C-CTXs and I-CTXs to their oxidized forms in fish tissues. C-CTXs, endemic in the Caribbean Sea, and a wide variety of different dinoflagellate species, were recently identified and confirmed in fish and waters from the western Atlantic Ocean, mainly near the Canary Islands (Spain) and Madeira archipelago (Portugal), which are therefore considered emerging areas for the occurrence of CP [12,13,14,15]. 

**Figure 1:**
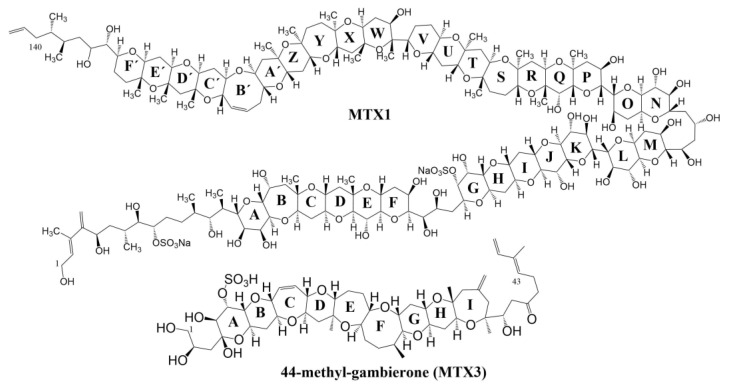
Structures of MTX1 and 44-methyl-gambierone (MTX3).

MTXs are considered the most toxic marine biotoxins identified to date [16]. Six MTX analogues have been identified to date: maitotoxin-1 (MTX1), maitotoxin-2 (MTX2), maitotoxin-3 (44-methyl gambierone), maitotoxin-4 (MTX4), desulfo-MTX1 and didehydro-demethyl-desulfo-MTX1 (Figure 1). All MTXs are characterized structurally by having at least one sulfate group, giving them increased polarity compared to CTXs. Their higher polarity limits their absorption when ingested, leading to them not being considered the main compounds responsible for CP, despite the presence of reports that suggest their accumulation in fish tissues [17]. MTX analogues are large natural non-polymeric compounds around 3300–3400 Da in size [18,19,20,21]. MTX1 and MTX4 have been associated with a massive calcium influx in cells, causing mortality. MTX2 was isolated in the 1990s [19,22], but its structure is still unknown. It is 1.6-fold less toxic than MTX1 by i.p. injection [23]. The structure of MTX3 was recently elucidated and corresponds to 44-methyl gambierone with a molecular weight of 1/3 MTX1 and has very low CTX-like activity, possibly related to the sulfate group in its structure (Figure 1) [24,25]. Therefore, the compound previously called MTX3 is referred to as 44-methyl gambierone rather than MTX3. However, recent studies suggest that MTX3 is not 44-methyl gambierone due to the low toxicity and inconsistent signs reported for 44-methyl gambierone compared with MTX3 [26]. Further research will be needed to confirm if these compounds, which might not be biologically equivalent, are the same molecule. Desulfo-MTX1 and didehydro-demethyl-desulfo-MTX1 were partially elucidated, being isolated from *G. belizeanus* strain CCMP 399 and *Gambierdiscus* ribotype-2 strain CCMP 1655, respectively, from the Caribbean Sea, and their biological activity is still unknown [21].

Surveillance programs are being globally implemented to evaluate the presence and spread of CP [27]. Concerning the European coasts, increased sampling of fish and dinoflagellates is being carried out, not only in emerging regions of the west Atlantic, such as the Canary Islands (Spain) and Madeira archipelago (Portugal), but also in the Mediterranean Sea where no cases of CP have yet been reported [28]. A number of dinoflagellate species have been recently identified in the Canary Islands (Spain), Selvagens Islands (Portugal), Mallorca (Spain), Menorca (Spain) and Crete (Greece) [29,30,31,32,33,34]. Initial characterization by LC-MS (both LC-MS/MS and LC-HRMS) of the toxins produced by these dinoflagellates has been recently carried out [33], but more research is needed to fully understand the differences in the toxicity profiles in relation to the geographical areas.

The present study focused on the characterization of a toxic strain of *G. australes* from the Mediterranean Sea (Menorca, Spain), with the aim of identifying the different compounds involved in the toxicity profile. This strain was previously characterized as having both CTX-like and MTX-like activity in the N2a. It has N2a and 44-methyl gambierone, and gambieric acids C and D, were discovered via LC-HRMS and LC-MS/MS [33,34]. However, MTX1, which is typically detected in some *G. australes* strains from the Pacific Ocean, was not detected, and the MTX-like toxicity detected in this strain was not associated with the presence of 44-methyl gambierone, which showed some CTX-like activity [25]. Therefore, an approach already used by the research team involved in this work for the characterization of CTXs in the absence of reference materials was also applied on this occasion to characterize the toxins involved in the toxicity profile [35]. The approach consisted of combining an initial fractionation by HPLC-C18, screening the toxicity of the fractions using N2a and selecting the toxic fractions to be further confirmed and characterized by LC-HRMS and LC-MS/MS. 

## 2. Results and Discussion

### 2.1. LC-MS/MS Analysis

**Figure 2:**
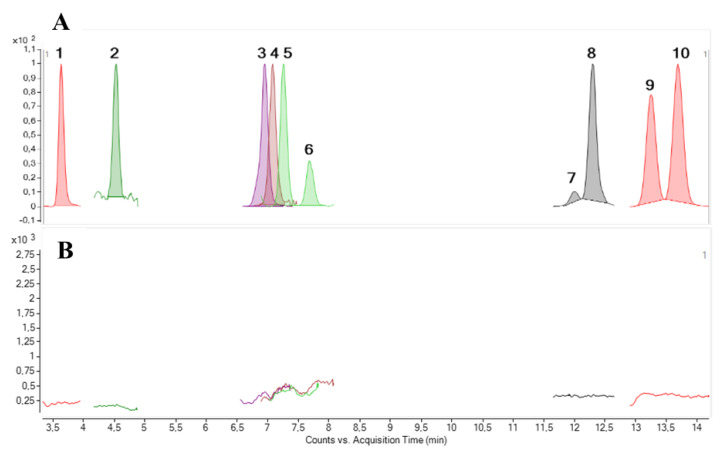
LC-MS/MS chromatogram of (**A**) the mixture of CTX reference materials: CTX1B (1), C-CTX1 (2), 2,3-dihydroxyCTX3C (3), 51-hydroxyCTX3C (4), 52-epi-54-deoxyCTX1B (5), 54-deoxyCTX1B (6), 49-epi-CTX3C (7), CTX3C (8), CTX4A (9) and CTX4B (10); (**B**) *G. australes* strain.

LC-MS/MS under the conditions described by Estevez et al. [36] was applied for the analysis of the crude methanolic extract obtained from the extraction of a *G. australes* (IRTA-SMM-17-271) strain from Menorca (Mediterranean Sea). CTXs were monitored selecting [M+Na]^+^ as the precursor and product ion in the Multiple Reaction Monitoring (MRM) mode and using the standards available: C-CTX1 and different P-CTXs analogues (CTX1B, 2,3-dihydroxyCTX3C, 51-hydroxyCTX3C, 52-epi-54-deoxyCTX1B, 54-deoxyCTX1B, 49-epi-CTX3C, CTX3C, CTX4A and CTX4B). The results obtained showed no presence of any of the CTX analogues monitored (LOD (S/N > 3) = 0.04 ng CTX1B/mL) (Figure 2).

As described above, the toxicity in this strain was recently evaluated with the N2a assay by Tudó et al. [34], which found MTX-like activity. However, previous studies showed that MTX1, which is typically detected in some *G. australes* strains from the Pacific Ocean, was not detected in this strain [33]. Therefore, the Find-by-molecular-feature^®^ algorithm of the LC-HRMS was used to identify the possible presence of an MTX analogue responsible for the toxicity observed in the N2a.

### 2.2. LC-HRMS Analysis

After liquid–liquid partitioning to selectively separate MTX-like compounds in the methanol soluble fraction (MSF) from CTX-like compounds in the dichloromethane soluble fraction (DSF), the *G. australes* sample was analyzed by LC-HRMS using the Find-by-molecular-feature^®^ algorithm. By using this algorithm, an unknown compound can be identified by restricting the retention time, *m/z*, ionization mode and ion species. The sample was analyzed in both negative and positive ionization modes using the LC-HRMS instrument (Agilent 6550 iFunnel Q-Tof) for full MS scanning.

#### 2.2.1. LC-HRMS in Negative Ionization Mode (ESI^−^)

**Figure 3:**
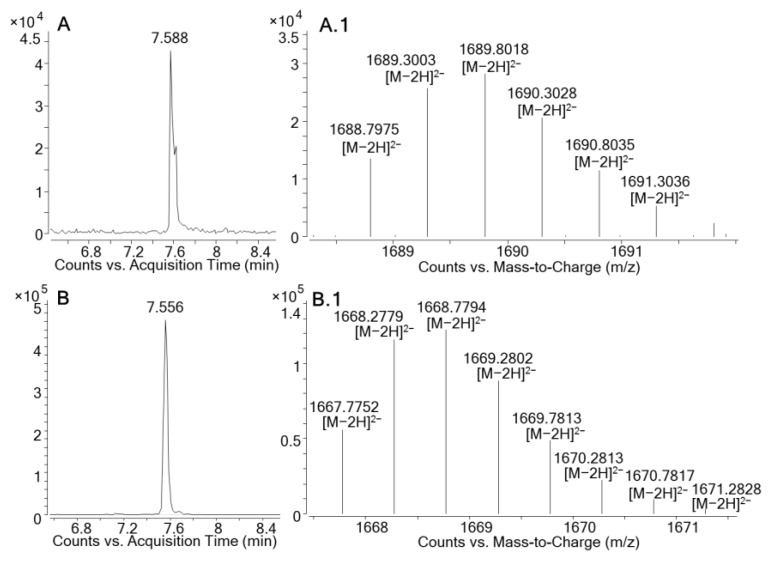
LC-HRMS chromatogram of (**A**) maitotoxin-1 standard (10 µg/mL); (**B**) the new congener of MTX1 detected in *G. australes*. HRMS spectra in negative full scan analysis of: (**A.1**) maitotoxin-1 standard (10 µg/mL); (**B.1**) the new congener of MTX1 detected in *G. australes*.

The negative ionization mode was considered the best approach to monitor for MTXs due to the lower matrix effect present during the ionization of these compounds. Not only was higher sensitivity compared to the positive ionization mode obtained in this mode, but also the ability to select the doubly-charged anion which was in the calibrated mass range of the mass spectrometer (50–3200 Da) was present. The Molecular-Feature-Extraction (MFE) algorithm provided in the Agilent MassHunter Qualitative Analysis allowed us to find compounds structurally similar to maitotoxin-1, with an *m/z* > 1500 and giving rise to a prominent doubly-charged anion (Δ*m/z* of 0.5 amu between the signals of the isotopic pattern in contrast with the mono-charged species in which Δ*m/z* is 1 amu). Results showed the presence of a prominent bicharged anion in the MSF, with a monoisotopic mass of *m/z* 1667.7752. This compound showed a retention time close to MTX1 (ΔRt= −0.032 min), indicating not only an isotope pattern similar to MTX1 but also nearly the same retention in the chromatographic separation using a C18 column (Table 1, Figure 3). Therefore, this compound, which was only detected in the MSF, appears to be structurally related to MTX1, taking into account the data obtained from the chromatography and mass spectrometry.

**Figure 4:**
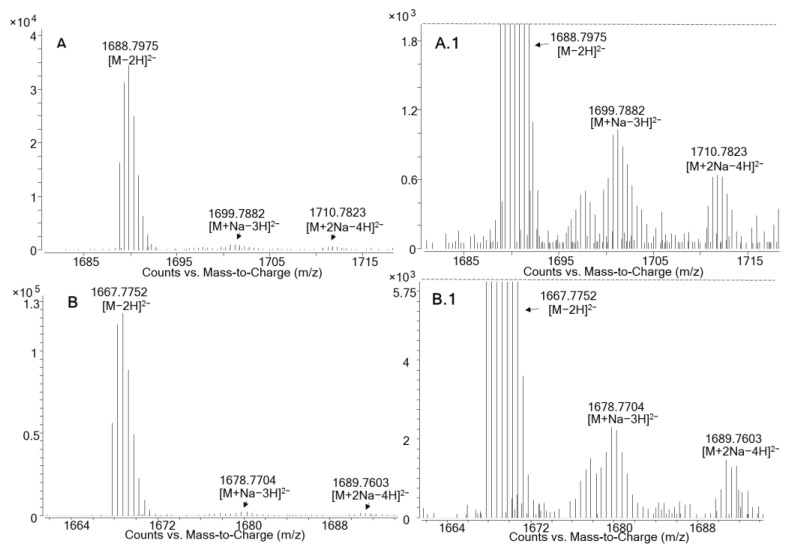
HRMS spectra in negative full scan mode of: (**A**) maitotoxin-1 standard (10 µg/mL); (**B**) the possible new maitotoxin analogue detected in *G. australes*. (**A.1**) Zoom of A; (**B.1**) zoom of B. The *m/z* values highlighted in the figure correspond to the measured monoisotopic masses.

MTX1 and the compound structurally related to MTX1, presumably a new maitotoxin analogue, showed the same ion clustering with a prominent doubly-charged anion [M−2H]^2−^. We found an accurate monoisotopic *m/z* of 1688.7975 (Δppm: −2.3) for MTX1, and one of 1667.7752 for the presumably new maitotoxin analogue; no triply [M−3H]^3−^ or quatruply-charged [M−4H]^4−^ ions were detected from either MTX1 or the possible new maitotoxin analogue, probably due to the lack of sensitivity for these particular ions. Traces of the ions [M+Na−3H]^2−^, *m/z* 1699.7882 (Δppm: −2.4) for MTX1 and *m/z* 1678.7704 for the new maitotoxin analogue, and [M+2Na−4H]^2−^, *m/z* 1710.7823 (Δppm: −0.6) for MTX1 and *m/z* 1689.7603 for the new maitotoxin analogue, were detected in both compounds with the same relative intensities (Figure 4). Maitotoxin-1 has a higher molecular weight (for the free acid form) compared to the new maitotoxin analogue—i.e., 3379.6172 versus 3337.5649.

#### 2.2.2. LC-HRMS in Positive Ionization Mode (ESI^+^)

**Figure 5:**
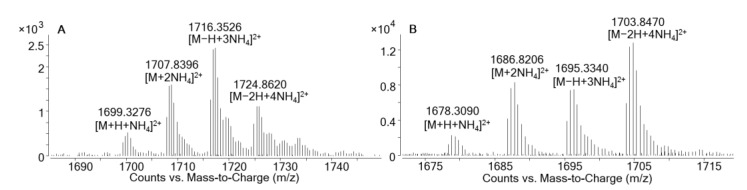
HRMS spectra in positive full scan mode of: (**A**) maitotoxin-1 standard (10 µg/mL); (**B**) the new maitotoxin analogue detected in *G. australes.* The *m/z* values highlighted in the figure correspond to the measured monoisotopic masses.

Positive electrospray HRMS full scan spectra were also obtained for both MTX1 and the new maitotoxin analogue. As reported in negative ionization mode, the singly charged molecule was not detected, most likely due to low abundance. Additionally, mass accuracy of the singly charged ion would be reduced, as it was out of the calibrated range of the mass spectrometer for this acquisition method (50–3200 Da). Therefore, doubly charged ions were monitored. Maitotoxin-1 showed ion clusters with ammonium adducts, i.e., the single adduct, *m/z* 1699.3276 [M+H+NH_4_]^2+^; double adduct, *m/z* 1707.8396 [M+2NH_4_]^2+^; triple adduct, *m/z* 1716.3526 [M−H+3NH_4_]^2+^; and quadruple adduct, *m/z* 1724.8620 [M−2H+4NH_4_]^2+^ (Table 1, Figure 5A). The same ion clusters were observed for the maitotoxin analogue but with different relative intensities. The quadruple ammonium adduct, *m/z* 1703.8470 [M−2H+4NH_4_]^2+^, was the most intense ion in comparison with MTX1, for which the triple ammonium adduct, *m/z* 1716.3526 [M−H+3NH_4_]^2+^, was most abundant. As indicated above, the sensitivity in positive ionization mode was around 10 times lower compared to the negative ionization mode (Table 1, Figure 5B).

The formation of the doubly charged ammonium adduct was previously reported by Mazzola et al. [21] for MTX1, desulfo-MTX1 and didehydro-demethyl-desulfo-MTX1, and for MTX4 by Pisapia et al. [37]. According to the nomenclature established for MTX analogues, this compound was therefore named MTX5.

#### 2.2.3. Molecular Formula Determination for MTX5

A molecular formula for MTX5 was proposed, as described in Pisapia et al. [37] and using the open source software ChemCal [38]. The Molecular Formula Finder application allowed us to obtain the most appropriate hypothetical molecular formula. From the HRMS spectra in negative and positive ionization modes, we confirmed a molecular mass of 3337.5649 (free acid form) for MTX5.

The modelling of MTX5’s molecular formula was based on its molecular mass of 3337.5649 (free acid form), and the structural similarities with MTX1 using the following assumptions: (1) a similar degree of unsaturation, ranging from 30 to 45, taking into account the similar molecular size of MTX1 and MTX5; (2) the numbers of atoms were: C: 100–200, H: 200–400, O: 50–150 and S: 1 or 2; and (3) the mass accuracy range was set to ±10 ppm.

The average of the absolute values of Δppm (|Δppm|) was calculated for each molecular formula proposed by ChemCal, taking into account the seven ion species of MTX5: [M−2H]^2−^, [M+Na−3H]^2−^, [M+2Na−4H]^2−^, [M−2H+4NH_4_]^2+^, [M−H+3NH_4_]^2+^, [M+2NH_4_]^2+^ and [M+H+NH_4_]^2+^ (Table S1). A total of 11 molecular formulae with average |Δppm| < 10 were considered as potential candidates, and the five molecular formulae with an average |Δppm| < 3 were chosen for further consideration (Table S1).

The experimental isotopic profiles of the most intense clusters of the positive and negative ionization modes ([M−2H]^2−^, [M−2H+4NH_4_]^2+^, [M−H+3NH_4_]^2+^ and [M+2NH_4_]^2+^) of MTX5 were compared with the theoretical ones simulated by ChemCal for the five molecular formulae with average |Δppm| < 3. The comparison of the isotopic profiles only allowed us to discriminate formulae #05, in which [M−2H]^2−^ showed Δppm > 4 (Table S2). In the remaining molecular formulae (#01–#04) the isotopic profiles were very similar. However, molecular formulae #01, #03 and #04 could be excluded due to the low (32 for #1) or high (41 for #03, and 45 for #04) degrees of unsaturation compared with MTX1 (Table S1). Additionally, the molecular formulae containing one sulfate atom (#01 and #03) seem unlikely compared to MTX1. 

Therefore, the molecular formula proposed for MTX5 was C_161_H_252_O_68_S_2_ (#02, average |Δppm| = 1.1, 36 degrees of unsaturation, Table S1). MTX5 would contain three carbons and six hydrogens less than MTX1, and the same numbers of oxygen and sulfur atoms and degrees of unsaturation as MTX1.

Further HRMS/MS data are needed to confirm the presence of two sulfate groups and to compare MTX5 and MTX1 fragmentation, but the lack of sufficient MTX5 reference material limited these experiments. Additionally, NMR and LC-HRMS analyses are needed to the complete the characterization of MTX5, but it is necessary to accumulate a large amount of biomass of the *G. australes* strain which currently is not available.

### 2.3. HPLC Fractionation 

The extract of *G. australes* strain IRTA-SMM-17-271, containing MTX5, was fractionated using an HPLC C18 column in order to isolate this compound and evaluate its response to the N2a cells. The HPLC C18 fractionation method coupled to the evaluation of the toxicity by N2a was previously applied to extracts of *G. excentricus* [20] and CP contaminated fish samples. C-CTX1 and three C-CTXs congeners were successfully isolated and detected as the main CTXs [35]. This approach of HPLC fractionation but coupled to the evaluation of the toxicity of *Gambierdiscus* through SH-SY5Y cell assay was also described in [39].

**Figure 6:**
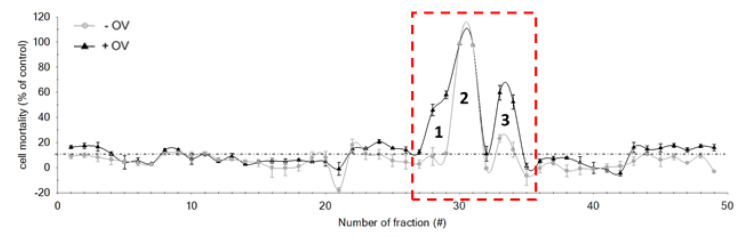
Cell mortality according to fraction number collected after HPLC-C18 fractionation. One hundred algae cells eq/well were added for each fraction. Presence (▲) and absence (•) of OV. The red box highlights the main toxic region, including the fractions ranging from 27 to 35. The dotted line shows a N2a cell survival ~90%; fractions nearby this line contained compounds with no detectable toxic activity.

The initial methanol crude extract of *G. australes* was fractionated to yield 49 fractions, all of which were screened with the N2a cell assay following the conditions described by Castro et al. [40] (Figure 6). No partitioning between DCM and aqueous methanol had been applied prior to the C18-HPLC fractionation, as a previous study indicated that several analogues, including gambierone and 44-methylgambierone, may partition into both fractions [33]. 

**Figure 7:**
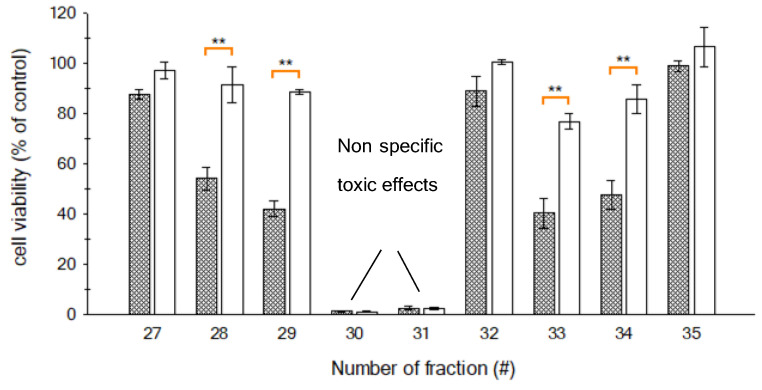
N2a cell viability of fractions included in the toxic region obtained from HPLC fractioning of *G. australes* crude extract, for sensitized (grey) and non-sensitized (white) cells. ** A fraction where a significant difference between sensitized and non-sensitized cells was observed (*t*-Student´s test, *n* = 3, *p* < 0.05).

After the N2a analysis of the 49 fractions, those producing mortality <10% were considered non-toxic, and this reduction in cell viability was associated with experimental variations. However, three peaks with toxic effects were detected. Peak 1 (fractions 28 and 29), showed CTX-like activity. It exhibited a reduction in cell viability in the sensitized cells (+OV); there was a statistically significant difference (*p* < 0.05) between the responses of sensitized and non-sensitized cells (Figure 7, Table 2). The most toxic effect was observed for peak 2 (fractions 30 and 31), with 100% mortality in both sensitized (+OV) and non-sensitized (–OV) cells. There was not a statistically significant difference between +OV and −OV cells (Figure 7, Table 2). Finally, for peak 3 (fractions 33 and 34), while inducing some −OV mortality (~30%), significantly increased N2a mortality after sensitization with ouabain and veratridine. Again, the responses of +OV and −OV cells statistically significantly different (*p* < 0.05) (Figure 7, Table 2). This slight reduction in −OV cell survivability may have been either due to the possible presence of compounds which did not act directly on site five of voltage-gated sodium channels (VGSC) or a more complex mechanism of the compound identified in this fraction.

The non-specific toxicity observed in peak 2 (Fractions 30 and 31) may theoretically have been caused by endogenous compounds of the matrix, or it could be associated with the presence of other toxins different than CTXs. However, due to the dilution of 1:180 of each fraction from the crude extract and the high toxicity observed, it is likely that this toxic effect was caused by other bioactive compounds known to be produced by *G. australes*, such as MTXs. Furthermore, the MTX-like toxicity reported in previous studies of this strain confirmed that the non-specific toxicity observed in peak 2 (fractions 30 and 31) corresponds to MTX-like toxicity [34].

### 2.4. LC-MS/MS Analysis of the Toxic Fractions

The method described by Estevez et al. [36] for the CTX analysis in fish tissue was adapted and used to monitor the presence of MTX5 and other toxic compounds in the fractions that showed toxic responses in the N2a assay.

**Figure 8:**
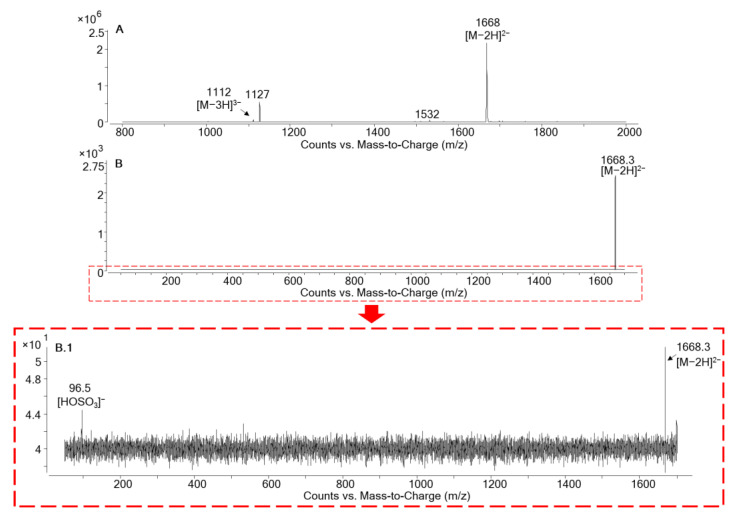
Mass spectra obtained by LC-MS/MS (Agilent 6495 iFunnel triple quad) in negative electrosrpay mode in the toxic fractions from peak 2 (fractions 30 and 31). (**A**) Full MS scan analysis obtained from *G. australes*. (**B**) Product ion selecting MTX5 *m/z* 1668 [M−2H]^2−^ at 70 eV. (**B.1**) Zoom of B and detection of traces of MTX5 hydrogen sulfate ion *m/z* 96.5 [HOSO_3_]^−^.

Full scan analysis of the main toxic fraction (peak 2, fractions 30 and 31) showed a prominent peak with *m/z* 1668 and traces of *m/z* 1112 both, assigned to MTX5’s doubly and triply-charged anions, [M−2H]^2−^ and [M−3H]^3−^. Unidentified masses with *m/z* 1532 and *m/z* 1127 were also detected, perhaps due to coeluting compounds (Figure 8A). A product ion scan of the highest intensity ion of MTX5, *m/z* 1668 [M−2H]^2−^, was carried out at different collision energies in order to detect the hydrogen sulfate anion loss *m/z* 96.5 [HOSO_3_]^−^, which is typical of MTXs and is used as a confirmatory ion for these compounds [20,41] (Figure 8B,B.1). Traces of the hydrogen sulfate anion were detected when applying 70 eV, confirming the high stability of MTX5 *m/z* 1668 [M−2H]^2−^, as has also been reported for MTX1 and MTX4 [20].

**Figure 9:**
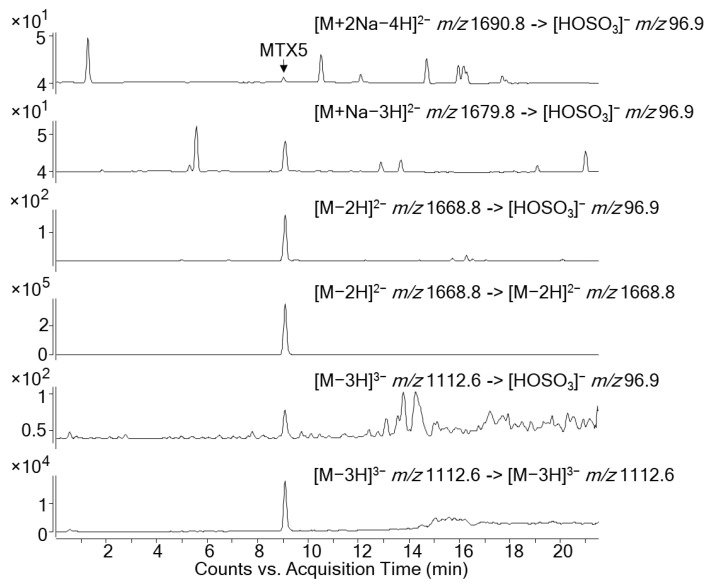
LC-MS/MS analysis in Multiple Reaction Monitoring Mode of MTX5 (9.05 min) detected in peak 2 (fractions 30 and 31) of *G. australes* after the HPLC fractionation.

A Multiple Reaction Monitoring (MRM) method was used to confirm the presence of MTX5 in the fractions that showed the non-specific toxicity. The MRM transitions in negative ionization mode were based on the method proposed by Pisapia et al. [20] for MTX1 and MTX4 (Table 2). The selection of the doubly charged anion [M−2H]^2−^ *m/z* 1668.8 of MTX5 as a precursor and product ion at 30 eV showed a prominent peak without fragmentation at a retention time of 9.05 min. The collision energy increase to 60 eV allowed us to monitor the hydrogen sulfate loss typical of MTXs through the transition [M−2H]^2−^ *m/z* 1668.8 -> [HOSO_3_]^−^ *m/z* 96.9. The same approach was carried out by selecting the triply-charged anion at the same retention time during the transitions [M−3H]^3−^ *m/z* 1112.6 -> [M−3H]^3−^ *m/z* 1112.6 and [M−3H]^3−^ *m/z* 1112.6 -> [HOSO_3_]^−^ *m/z* 96.9. Further confirmation of the presence of MTX5 was carried out by monitoring the hydrogen sulfate loss with precursor ions [M+Na−3H]^2−^ *m/z* 1679.8 and [M+2Na−4H]^2−^ *m/z* 1690.8 (Figure 9). These results show that this novel MTX was present in the fractions showing non-specific toxicity (peak 2, fractions 30 and 31). However, the novel MTX analogue responsible for the toxic activity of these fractions should be considered preliminary, and further experiments are needed to selectively isolate this compound in a pure fraction. The lack of enough *G. australes* biomass, and thereby isolated MTX5, limited the performance of additional HRMS analysis. Future work will be focused on the complete characterization of MTX5 by its isolation and structural elucidation by HRMS and NMR.

Further characterization of the fractions toxic in the N2a (peak 1, fractions 28 and 29; and peak 3, fractions 33 and 34) was carried out. For this purpose, an LC-MS/MS method, with screening and confirmation purposes, was proposed based on the conditions described by Estevez et al. [36], initially developed for the confirmation of C-CTX1 in fish tissue. 

**Figure 10:**
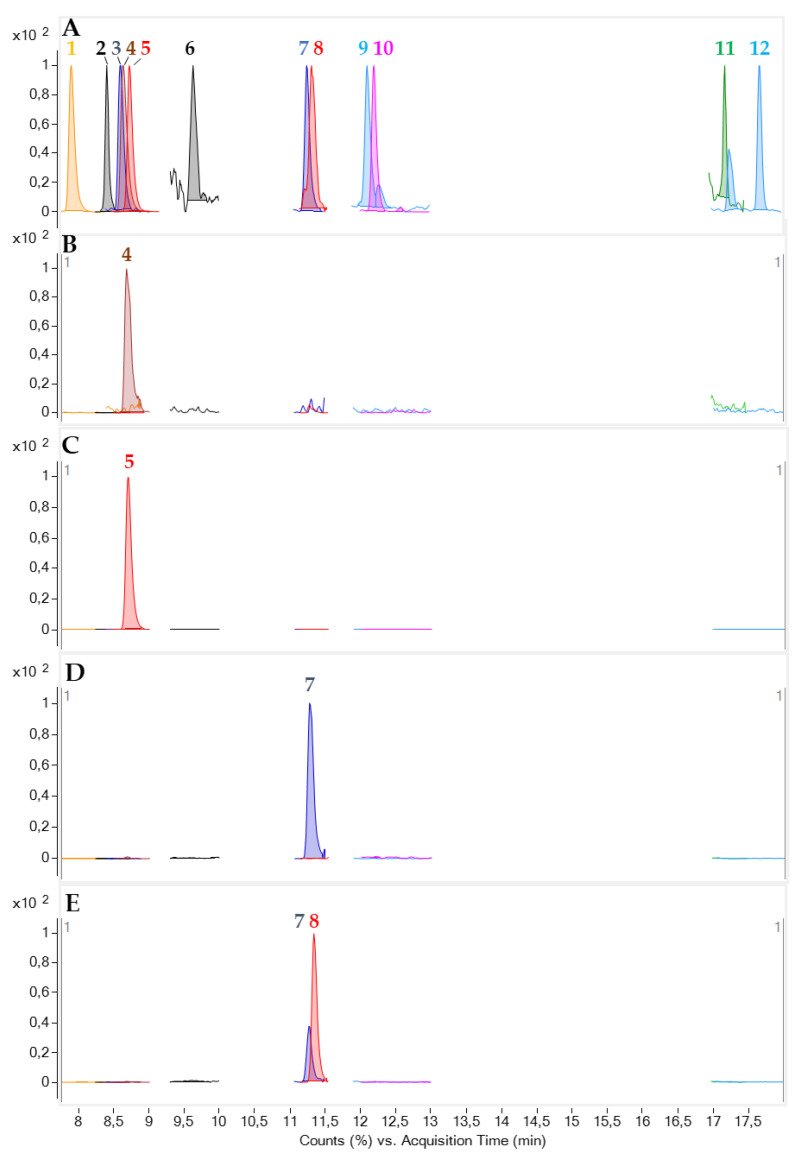
LC-MS/MS analysis in MRM mode showing the most abundant ion transition for each compound. (**A**) Reference material containing: gambierone (1), MTX4 (2), CTX1B (3), 44-methyl gambierone (4), MTX5 (5), C-CTX1 (6), gambieric acid C (7), gambieric acid D (8), 51-hydroxyCTX3C (9), 52-*epi*-54-deoxyCTX1B/54-deoxyCTX1B (10), CTX3C (11), CTX4A/CTX4B (12); (**B**) 44-methyl gambierone (4) detected in fraction 28; (**C**) MTX5 (5) detected in fraction 30. (**D**) Gambieric acid C detected in the fraction 33; (**E**) Coelution of gambieric acids C and D in the fraction 34.

The availability of a mixture of different toxic metabolites produced by dinoflagellates (gambierone, MTX4, 44-methyl gambierone, gambieric Acid C and gambieric acid D), fish and algal *p*-CTXs (CTX1B, 51-hydroxyCTX3C, 52-*epi*-54-deoxyCTX1B, 54-deoxyCTX1B, CTX3C, CTX4A and CTX4B) and C-CTX1, allowed us to evaluate the presence of these compounds in the different toxic fractions. The MRM method was based not only on the ion transitions reported in the bibliography for the different toxic compounds, but also on the retention time by comparing the standards and reference materials available, allowing their unequivocal confirmation (Figure 10A) [33] (see detailed MRM transitions in Table S4). 

Gambierone and 44-methyl gambierone were analyzed in positive and negative ionization modes with fast polarity switching. Negative ionization mode monitored not only the [M−H]^−^ as a precursor and product ion, but also the loss of the hydrogen sulfate ion [HOSO_3_]^−^ *m/z* 96.9 typical of these compounds. On the other hand, positive ionization mode monitored [M+H]^+^ as a precursor ion, and as product ions common fragments such as *m/z* 803 and *m/z* 109, and fragments *m/z* 219.1 and *m/z* 233.1 specific for gambierone and 44-methyl gambierone, respectively [25,33].

Negative ionization mode was used to monitor MTX4 by selecting its doubly charged anion as the precursor and product ion *m/z* 1646.2 [M−2H]^2−^ > *m/z* 1646.2 [M−2H]^2−^, and the hydrogen sulfate anion loss *m/z* 96.9 [HOSO_3_]^−^ [20].

Gambieric acids C and D (GAC and GAD) were also monitored in both negative and positive ionization modes, based on the conditions proposed by Estevez et al. [33]. The negative ionization mode was based on the selection of precursor and product ions of [M−H]^−^. Positive ionization mode monitored as a precursor ion [M+H]^+^_,_ and as specific ions product ions *m/z* 1039.6 and *m/z* 943.5 for GAC, and *m/z* 1053.6 and *m/z* 957.6 for GAD. The common product ion monitored for both compounds was *m/z* 135.1.

CTX1B was monitored by selecting the ammonium adduct as the precursor ion *m/z* 1128.6 [M+NH_4_]^+^, monitoring the first water loss *m/z* 1093.6 [M+H−H_2_O]^+^ and specific ions reported in the bibliography *m/z* 171.2 and *m/z* 95.2 from the fragmentation in CTX1B A-ring [42,43,44]. The same approach of monitoring the first water loss [M+H−H_2_O]^+^ and two specific ions *m/z* 155.1 and *m/z* 125.1 from fragmentation of the K, L and M rings, was carried out for 52-*epi*-54-deoxyCTX1B, 54-deoxyCTX1B, 51-hydroxyCTX3C, CTX3C, CTX4A and CTX4B [42,43,44,45].

C-CTX1 was monitored according to the conditions described by Estevez et al. [36] by selecting the first water loss precursor ion of *m/z* 1123.6 [M+H−H_2_O]^+^ and three water losses through the product ions and specific fragments *m/z* 191.1 and *m/z* 108.9. C-CTX1 methoxy congener, C-CTX1-Me, was also monitored in order to detect the possible degradation of C-CTX1 in the case of being present in the dinoflagellates samples [46].

The fractions where N2a toxicity was detected, fractions 28 and 29 (peak 1) and fractions 33 and 34 (peak 3), were analyzed with the MRM method previously described. The presence of 44-methyl gambierone was confirmed in fractions 28 and 29, suggesting its contribution to the N2a toxicity. Retention time and ion transitions and ion ratios were consistent with those obtained in the reference material (Figure 10A,B). As reported by Boente-Juncal et a. [25], 44-methyl gambierone shows some toxicity to N2a cells which may at least in part explain the toxicity observed in fractions 28 and 29.

GAC was tentatively confirmed in fraction 33 (peak 3), and a mixture of GAC and GAD was detected in fraction 34 (peak 3) (Figure 10A and D). Retention times and ion ratio transitions were the same as were obtained in the reference material. Despite the fact that GAs C and D are known to be potent antifungal agents, their biological activity is not clear, being mixtures of compounds that are moderately cytotoxic in mouse lymphoma cells L5178Y [47]. This study suggests that GAs C and D may contribute to activity towards VGSCs of the N2a cells. This result is in agreement with the biological activity of GAs A and B, which are inhibitors of brevetoxins (PbTx) through competitive binding on the 5-sides of the VGSCs [48]. However, the reduction in the cell viability in the unsensitized cells (–OV) suggested that these compounds might act over other channels and not exclusively on the VGSCs.

The toxicity profile of *G. australes* from the Mediterranean Sea is different to that observed in the same species in the Pacific Ocean. *G. australes* from the Pacific Ocean usually produces MTX1 and 44-methyl gambierone, and the same species in the Mediterrranean Sea produced MTX5 and 44-methyl gambierone and gambieric acids C and D [17,24]. This species seems to produce a large molecular weight MTX with MTX-like activity depending on the geographical region, and 44-methyl gambierone is a compound with CTX-like activity, the presence of which appears to be independent of the geographical region. In contrast, *G. excentricus* from the Canary Islands (Spain) only produced a large MTX compound, MTX4, with 44-methyl gambierone not being present [33].

The lack of additional fractions with CTX-like activity is remarkable and may be attributed to the presence of CTX precursors with little activity. Indeed, these precursors may be present in far lower amounts compared to MTXs or Gas, though these CTX precursors did not show any toxic activity in the N2a cells. A possible coelution of the CTX precursors with the MTX5 fraction covering up the CTX-like activity should be also explored.

Finally, this study was limited not only by the lack of enough biomass of the *G. australes* strain to perform additional analyses, but also of reference materials of MTXs. Therefore, experiments were focused on the identification of this novel MTX analogue. We combined multiple approaches, such as LC-MS (MS/MS and HRMS) and N2a guided LC-fractionation. The combination of all these approaches allowed the preliminary confirmation of a novel MTX analogue, and further work will be carried out to fully characterize this compound.

## 3. Materials and Methods 

### 3.1. Reference Materials and Chemicals

Gambierone detected in *Gambierdiscus sp2* from Crete (Greece), MTX4 detected in *G. excentricus* from the Canary Islands (Spain) and 44-methyl gambierone, gambieric acid C and D detected in *G. australes* from the Balearic Islands (Spain) were identified and confirmed by LC-HRMS and LC-MS/MS in previous studies; and a mixture of these compounds, and P-CTXs and C-CTX1, was used as a qualitative laboratory reference material [33]. 

The P-CTXs (CTX1B, 51-hydroxyCTX3C, 52-*epi*-54-deoxyCTX1B, 54-deoxyCTX1B, CTX3C, CTX4A and CTX4B) included in the qualitative laboratory reference material described above were kindly provided by Prof. Yasumoto (Japan Food Research Laboratory, Tokyo, Japan).

C-CTX1 qualitative laboratory reference material was also isolated and purified in previous work carried out by some of the authors of this study [35].

Maitotoxin-1 (MTX1) standard (10 µg mL^−1^) used in the LC-HRMS analysis was obtained from Wako Chemicals USA, Inc. (Richmond, VA, USA). 

Dichloromethane and methanol for the liquid–liquid partition, and methanol, ammonium formate and formic acid (Merck KGaA, Darmstadt, Germany) for HPLC-C18 fractionation were of HPLC grade. Acetonitrile, ammonium formate, formic acid, (Merck KGaA, Darmstadt, Germany) and water (J. T. Baker, Center Valley, PA, USA) for the LC-MS analysis were of LC-MS grade. H_2_O for dinoflagellate fractionation was deionized and purified at 15 M Ω cm^−1^ using a Milli-Q Gradient A10 system (Millipore, Spain). 

### 3.2. Gambierdiscus Australes Strain

The extract of the *G. australes* strain IRTA-SMM-17-271 was supplied by IRTA (Spain), through the EuroCigua project [49]. *G. australes* was collected in Macarella in Menorca Island (Balearic Islands, Macarella, Spain) and was cultivated at IRTA Marine and Continental Waters laboratory (Tarragona, Spain). The toxin profile and toxicity of this strain had been previously characterized by N2a and LC-MS (MS/MS and HRMS), and the results were reported in [33,34].

### 3.3. Sample Pretreatment

The methanol extract containing the equivalent of 1 million cells of *G. australes* (IRTA-SMM-17-271) was evaporated to a solid residue under N_2_ at 50 °C. The solid residue was reconstitued in dichloromethane and partitioned twice with MeOH:H_2_O (3:2) [50]. Both fractions were evaporated to dryness under reduced pressure in a rotatory evaporator at 45 °C. The dichloromethane soluble fraction (DSF), containing the CTX-like compounds, was reconstituted in 0.5 mL of MeOH, and the methanol soluble fraction (MSF), containing the MTX-like compounds, was reconstituted in 0.5 mL of MeOH:H_2_O (1:1). Both were filtered through 0.22 µm filters before being analyzed by LC-HRMS.

### 3.4. HPLC-C18 Fractionation

HPLC-C18 fractionation was carried out according to the conditions described in [35]. Briefly: The fractionation of extracts of dinoflagellates was carried out on an Agilent 1100 G1312A LC system coupled to an Agilent 1260 II automatic fraction collector with an Agilent 1260 II UV detector (Agilent Technologies, Waldbronn, Germany). A Kinetex® LC-C18 column (4.6 × 250 mm, 5 µm, 100 Å, Phenomenex) was used for the fractionation. Water with 5 mM of ammonium formate and 0.1% of formic acid (A) or methanol (B) constituted each mobile phase. The mobile phase gradient started from 60% B to 100% B, taking 85 min at a flow rate of 1 mL/min. The injection volume was 100 µL. A total of 49 fractions were collected. The solvent was removed by evaporation under N_2_ at 50 °C and reconstituted in 1 mL of MeOH LC-MS.

The initial methanolic extract of the *G. australes* (IRTA-SMN-17-271) was fractionated under the above-described conditions. A portion (1 mL) of the initial methanol extract, containing the equivalent of 609,011 cells, was evaporated to dryness under N_2_ at 50 °C and reconstituted in 100 µL of methanol and filtered through 0.22 µm (Syringe Driver filter Unit, Millex®-CV 0.22 um, 13 mm, Millipore, Billerica, MA, USA) before its injection into the HPLC system. 

### 3.5. N2a Assay

An N2a cell-based assay (CBA) was performed according to the conditions described in [40,51] with modifications to improve sensitivity and to be applied to the *G. australes* analysis.

N2a cell line was obtained from American Type Culture Collection (ATCC, CCL 131, Manassas, VA, USA) and cultured in 30 mL of growth medium consisting of RPMI-1640 medium (R8758, Sigma, Irvine, UK) containing 1% (*v/v*), 100 mM sodium pyruvate (S8636, Sigma, Irvine, UK), 1% (*v/v*) 200 mM L-glutamine (G7513, Sigma, Irvine, UK) and 1% (*v/v*) penicillin–streptomycin solution formed by 5000 units and 5 mg/mL, respectively (P4458, Sigma, St. Louis, MO, USA). This medium was supplemented with 10% (*v/v*) fetal bovine serum (FBS, F2442, Sigma, St. Louis, MO, USA) to obtain a complete growth media (RPMI-10). N2a cell line was maintained at 37 °C under 5% CO_2_ in a humidified atmosphere and sub-cultured when a confluence >80% was observed.

N2a cells were seeded into 96-well assay plates (Corning, NY, USA) at a density of 40,000 cells per well in 0.2 mL of growth medium supplemented with 5% (*v/v*) FBS (RPMI-5) and incubated for 24 h, at 37 °C, in a humidified atmosphere enriched with 5% of CO_2_. Plates were divided into non-sensitized and sensitized sections in order to detect the presence of CTX-like compounds. Cells from sensitized section were exposed to a mixture of 3 10^−3^ mM of ouabain (O3125, Sigma, St. Louis, MO, USA) and 3 10^−4^ mM of veratridine (V5754, Sigma, St. Louis, MO, USA) (+OV section), allowing a reduction of 20% of cell viability. Next, 20 µL of PBS was added to wells of the non-sensitized cells (−OV section) to match volumes between both sections.

Microalgae extracts from HPLC fractionation were first evaporated to dryness under N_2_ stream at 40 °C, and then reconstituted in 1 mL of MeOH (LC-MS grade). The optimal number of microalgae cell equivalents per well of each fraction was evaluated in order to obtain the best sensitivity avoiding matrix effects. The best results were obtained by diluting the crude extract from the fractionation to 1:180, which corresponds to the addition of 100 cells equivalents per well. In total, 10 µL per well of each fraction dilution was added, containing 20% MeOH, and each fraction was tested in triplicate on sensitized (+OV) and non-sensitized cells (−OV). To match the volume, 10 µL of RPMI-5 with 20% MeOH was added to control wells.

C-CTX1 standard solution was serially diluted ten times—50 to 0.5 pg·mL^−1^—to evaluate the sensitivity of the N2a cells to the presence of CTX-like compounds. N2a cells exhibited a toxic effect in the presence of ouabain/veratridine (OV), showing a sigmoidal dose–response curve. The inhibitory concentration at 50% (IC50) was 1.56 ± 0.11 pg C-CTX1 mL^−1^ (*n* = 4). The limit of detection (LOD) was 7.4 ± 1.2 fg C-CTX1 algae cell eq^−1^. 

After 16 h of incubation, cell viability was assessed by colorimetric MTT (3-(4,5-dimethylthiazol-2-yl)-2,5-diphenyl tetrazolium bromide, Sigma, St. Louis, MO, USA) method [52]. Briefly, the medium was removed from the wells and replaced by 60 µL of RPMI-5 containing 0.8 mg mL^−1^ of MTT. After 40 min at 37 °C, MTT medium was discarded, and the resulting formazan crystals, produced from mitochondrial dehydrogenases of living cells, were solubilized with 100 µL of dimethyl sulfoxide (DMSO, D5879, Honeywell, Seelze, Germany). Absorbance was measured with a spectrophotometer (Multiskan® FC Microplate Photometer, Thermo Fisher Scientific Oy, Ratastie, Finland) at 570 nm for testing and 630 nm for reference [52,53]. 

Data were processed by using SigmaPlot v.12.0 software. C-CTX1 standard was fitted to a sigmoidal dose-response curve and the IC_50_ (inhibition concentration causing a 50% reduction in the cell viability) was calculated using a four-parameter logistic function (4PL) with variable slope. Limit of detection (LOD) was calculated through the ratio between the IC20 of C-CTX1 standard (inhibition concentration causing a 20% reduction in the cell viability) and the number of algal cell equivalents added per well. Students t-tests were used to evaluate statistically significate differences between having and not having the OV treatment. Significant differences were admitted between +OV and −OV when *p* values were <0.05 at a 95% confidence level. 

### 3.6. LC-HRMS (Agilent 6550 Q-Tof iFunnel)

LC-HRMS analyses were performed following the conditions described by Estevez et al. [33] using a UHPLC system 1290 Infinity II (Agilent Technologies, Santa Clara, CA, USA) coupled to a quadrupole-time of flight mass spectrometer Q-Tof 6550 iFunnel (Agilent Technologies, Santa Clara, CA, USA). A Kinetex C18 column (100 Å, 1.7 µm, 100 × 2.1 mm, Phenomenex, Le Pecq, France) set at 40 °C was used for the chromatographic separation with a solvent system containing water (Mobile phase A) and 95% acetonitrile (mobile phase B); both contained 2 mM of ammonium formate and 50 mM of formic acid. The injection volume was 5 µL and the flow rate 0.4 mL·min^−1^. The chromatographic separation was carried out as follows: starting at 5% B and keeping it isocratic for 1 min; increasing to 100% B in 11 min; maintaining for 2 min; returning to 5% B in 0.5 min. The column was equilibrated for 4.5 min before injecting the next sample.

The LC-HRMS instrument operated in both negative and positive ionization full MS scan modes and was calibrated using the Agilent tuning mix before each analysis. Source and interface conditions were: drying gas, 11 L·min^−1^ at 160 °C; sheath gas flow, 11 L·min^−1^ at 250 °C; nebulizer, 45 psi; capillaries, (+) 4500 and (−) 4500 V; nozzles, (+) 500 and (−) 500 V. 

Full MS scan analysis operated at a mass resolution of 40,000 full width at half maximum (FWHM) over a mass-to-charge ratio (*m/z*) ranging from 100 to 3200, with a scan rate of 1 spectrum/s. The reference *m/z* 68.9958, 966.0007, 1865.9430 and 2465.9049 were continuously monitored during the entire run in negative ionization mode; and *m/z* 121.0509 and 922.0098 were monitored in positive ionization mode. Raw data were processed with Agilent MassHunter Qualitative Analysis software (version B.07.00, service pack 1, Santa Clara, CA, USA) using the Find by Formula (FbF) algorithm for MTX1. The Molecular Feature Extraction (MFE) algorithm (*m/z* > 1500, giving rise to doubly-charged anion [M−2H]^2−^ and a limit for the background noise set to 1000 counts) was used for MTX5.

### 3.7. LC-MS/MS (Agilent 6495 QQQ iFunnel)

LC-MS/MS analyses were carried out following the conditions described by Estevez et al. [36] with modifications. An Agilent 1290 Infinity LC system (Agilent Technologies, Santa Clara, CA, USA) coupled to an Agilent 6495 iFunnel triple quad (Agilent Technologies, Santa Clara, CA, USA). Two different methods were used:

The first method was used to analyze the crude extract of the *G. australes* strain. Briefly, the chromatographic separation was performed using a Poroshell 120 EC-C18 column (3.0 × 50 mm, 2.7 µm, Agilent, Santa Clara, CA, USA) at 40 °C. Mobile phase was H_2_O with 5 mM of ammonium formate and 0.1% of formic acid (A) or methanol (B). The mobile phase gradient started at 78% B; increased to 88% B at 10 min; held for 5 min at 88% B; increased at 15.01 min to 100% B; held until 18 min; returning to 78% B at 18.01 min. Equilibration took place for 4 min prior to the next injection. Injection volume was 1 µL, and the flow rate was 0.4 mL·min^−1^. The mass spectrometer operated in positive ionization using the Multiple Reaction Monitoring (MRM) mode. The detailed MRM transitions are shown in Table S3.

Source parameters were: drying gas, 15 L·min^−1^ at 290 °C, sheath gas flow 12 L·min^−1^ at 400 °C, nebulizer 50 psi, capillary (+) 5000 V, nozzle (+) 300 V and fragmentor voltage 380 V.

The second method was used to confirm the presence of toxic compounds in the fractions with CTX- and MTX-like toxicity. Briefly, the chromatographic separation was performed using a Poroshell 120 EC-C18 column (2.1 × 100 mm, 2.7 µm, Agilent, Santa Clara, CA, USA) at 40 °C. Mobile phase was H_2_O with 5 mM of ammonium formate and 0.1% formic acid (A) or acetonitrile (B). The mobile phase gradient started at 35% B for 2 min; increased to 80% B over 15 min; increased to 95% B over 1 min; held for 5 min; returned to 35% B at 21.5·min. Equilibration occurred 4 min prior to the next injection. Injection volume was 5 µL, and the flow rate 0.4 mL·min^−1^. The mass spectrometer operated in fast polarity switching in Multiple Reaction Monitoring (MRM) mode. The detailed MRM transitions are shown in Table S4. 

Source parameters were: drying gas, 16 L·min^−1^ at 250 °C; sheath gas flow, 12 L·min^−1^ at 400 °C; nebulizer, 15 psi; capillaries, (+) 5000 V and (−) 4500; nozzles, (+) 1000 V and (−) 1700; fragmentor voltage 380 V.

## 4. Conclusions

This work shows the potential of using the approach of combining HPLC fractionation with an N2a assay and mass spectral analysis as a tool for the identification and confirmation of CTX-like compounds when no standards are available. The results obtained using this combined approach allowed the identification for the first time of the presence of a new MTX analogue—MTX5—in an extract of *G. australes* from the Balearic Islands. MTX5 showed a retention time, molecular weight and ion pattern similar to MTX1, the latter being present as a hydrogen sulfate anion typical of MTXs. The comparison of MTX1 and MTX5 ion cluster profiles of the doubly charged ions of full scan positive and negative ionization mode allowed us to propose the molecular formula for MTX5 (C_161_H_252_O_68_S_2_). Further structural elucidation by HRMS and NMR is required for the complete characterization; however, the lack of biomass and reference materials has limited this task. The above-mentioned combined approach also allowed the confirmation of the presence of other CTX-like compounds that had been identified in previous work carried out with the same extracts. The presence of 44-methyl gambierone was confirmed in fractions with N2a activity in the *G. australes* strain. GAs C and D also appear to contribute to N2a activity. Further research is also needed to confirm whether these compounds directly act in a CTX-like fashion on VGSCs and may thus contribute to ciguatera poisoning.

## Figures and Tables

**Table 1:** Ions assigned to MTX1 standard and the new maitotoxin analogue detected in the *G. australes* (IRTA-SMM-17-271) from the Mediterranean Sea by ESI^−^ LC-HRMS. *m/z* values correspond to the accurate monoisotopic masses.

			MTX1		New Maitotoxin Analogue
Molecular Formula (M)			C_164_H_258_O_68_S_2_		Unknown
Relative Molecular Weight (g/mol)			3379.6172		3337.5649
Retention Time (min)			7.6		7.6
Ion Species (Monoisotopic Mass)			Theoretical	Measured	Measured
	ESI^−^	[M−2H]^2^^−^	1688.8013	1688.7975 (Δppm: −2.3)	1667.7752
		[M+Na−3H]^2^^−^	1699.7923	1699.7882 (Δppm: −2.4)	1678.7704
		[M+2Na−4H]^2^^−^	1710.7833	1710.7823(Δppm: −0.6)	1689.7603
		[M−3H]^3^^−^	1125.5318	n.d.	n.d.
		[M−4H]^4^^−^	843.8970	n.d.	n.d.
	ESI^+^	[M−2H+4NH_4_]^2+^	1724.8695	1724.8620 (Δppm: −4.4 )	1703.8470
		[M−H+3NH_4_]^2+^	1716.3562	1716.3526 (Δppm: −2.1)	1695.3340
		[M+2NH_4_]^2+^	1707.8430	1707.8396 (Δppm: −2.0)	1686.8206
		[M+H+NH_4_]^2+^	1699.3297	1699.3276 (Δppm: −1.2)	1678.3090

**Table 2:** MRM transitions for MTX5 monitored by the LC-MS/MS instrument.

Compound	Retention Time (min)	ESI	MRM Transitions Q1/Q3 (*m/z*)		CE (eV)	CAV (eV)
**MTX5**	9.05	-	[M−2H]^2−^/[M−2H]^2−^	1668.8/1668.8	30	5
			[M−2H]^2−^/[HOSO_3_]^−^	1668.8/96.9	60	5
			[M−3H]^3−^/[M−3H]^3−^	1112.6/1112.6	30	5
			[M−3H]^3−^/[HOSO_3_]^−^	1112.6/96.9	60	5
			[M+Na−3H]^2−^ /[HOSO_3_]^−^	1679.8/96.9	60	5
			[M+2Na−4H]^2−^ /[HOSO_3_]^−^	1690.8 /96.9	60	5

## Supplementary Materials

The following are available online at https://www.mdpi.com/article/10.3390/md19080460/s1. Table S1. List of the 11 molecular formulae (M, as unsalted molecule). Unsaturations generated by ChemCalc for MTX5. Mass differences (Δ ppm) between the accurate monoisotopic *m/z* of MTX5 spectra and the theoretical *m/z*. Ranking was based on the averages of the absolute values of Δ ppm (|Δ ppm|). M were excluded from further studies because they presented average |Δ ppm| > 3 (grey). Proposed M for MTX5 (black). Table S2. Isotopic ratio profiles obtained from experimental data of MTX5 and from theoretical data of the selected molecular formulae (#01, #02, #03, #04 and #05, Table S1). Most intense ions species of the positive and negative HRMS spectra of MTX5. RA: Relative abundance. Table S3. MRM ion transitions of the different CTXs monitored by LC-MS/MS. Table S4. MRM ion transitions monitored by LC-MS/MS of the different toxic metabolites produced by dinoflagellates and accumulated in fish tissue.
