# Supplementary material for: Toxicity Screening of a Gambierdiscus australes Strain from the Western Mediterranean Sea and Identification of a Novel Maitotoxin Analogue
Source: Mar Drugs. 2021 Aug 15;19(8):460. doi: 10.3390/md19080460 (PMC8400318; doi:10.3390/md19080460)
Supplement: Supplementary file 1 [file marinedrugs-19-00460-s001.zip › marinedrugs-1319979-supplementary.pdf]

Supplementary materials : Toxicity screening of a *Gambierdiscus australes* strain from the western Mediterranean Sea and identification of a novel Maitotoxin analogue

Pablo Estevez, David Castro, José Manuel Leão-Martins, Manoëlla Sibat, Angels Tudó, Robert Dickey, Jorge Diogene, Philipp Hess, Ana Gago-Martinez

Table S1. List of the 11 molecular formula (M, as unsalted molecule). Unsaturations generated by ChemCalc for MTX5. Mass differences (Δ ppm) between the accurate monoisotopic *m/z* of MTX5 spectra and the theoretical *m/z*. Ranking was based on the average of the absolute values of Δ ppm (|Δ ppm|). M excluded from further studies because they presented an average |Δ ppm| > 3 (grey). Proposed M for MTX5 (black).

Ranking	Molecular formula (M)	Unsaturations	|Δ ppm| Average	Δ ppm						
				$[M-2H]^{2-}$	$[M+Na-3H]^{2-}$	$[M+2Na-4H]^{2-}$	$[M-2H+4NH_4]^{2+}$	$[M-H+3NH_4]^{2+}$	$[M+2NH_4]^{2+}$	$[M+H+NH_4]^{2+}$
#01	$C_{157}H_{252}O_{73}S$	32	0,7	-2,3	0,2	-0,4	0,2	0,3	0,2	1,3
#02	$C_{161}H_{252}O_{68}S_2$	36	1,1	-1,6	1,0	0,3	0,9	1,1	1,0	2,0
#03	$C_{164}H_{248}O_{68}S$	41	1,8	-0,6	2,0	1,3	1,9	2,1	2,0	3,0
#04	$C_{168}H_{248}O_{63}S_2$	45	2,4	0,2	2,7	2,0	2,6	2,8	2,7	3,8
#05	$C_{168}H_{248}O_{65}S$	45	2,7	-5,2	-2,6	-3,2	-2,6	-2,5	-1,7	-1,5
#06	$C_{165}H_{252}O_{65}S_2$	40	3,9	-6,2	-3,6	-4,2	-3,6	-3,5	-3,6	-2,6
#07	$C_{161}H_{252}O_{70}S$	36	4,6	-6,9	-4,3	-5,0	-4,3	-4,2	-4,3	-3,3
#08	$C_{157}H_{252}O_{71}S_2$	32	5,2	3,0	5,5	4,8	5,3	5,5	5,5	6,6
#09	$C_{158}H_{256}O_{70}S_2$	31	5,6	-7,9	-5,4	-6,0	-5,3	-5,2	-5,3	-4,3
#10	$C_{160}H_{248}O_{71}S$	37	6,2	4,0	6,5	5,8	6,3	6,5	6,5	7,5
#11	$C_{164}H_{248}O_{66}S_2$	41	6,9	4,7	7,2	6,6	7,1	7,3	7,2	8,3

Table S2. Isotopic ratio profiles obtained from experimental data of MTX5 and from theoretical data of the selected molecular formulae (#01, #02, #03, #04, and #05, Table S1). Most intense ions species of the positive and negative HRMS spectra of MTX5. RA: Relative abundance.

Ion species	MTX5 Experimental data		(#01) $C_{157}H_{252}O_{73}S$			(#02) $C_{161}H_{252}O_{68}S_2$			(#03) $C_{164}H_{248}O_{68}S$			(#04) $C_{168}H_{248}O_{63}S_2$			(#05) $C_{168}H_{248}O_{65}S$		
	m/z	RA(%)	Theoretical *m/z*	Δppm	RA (%)	Theoretical *m/z*	Δppm	RA (%)	Theoretical *m/z*	Δppm	RA (%)	Theoretical *m/z*	Δppm	RA (%)	Theoretical *m/z*	Δppm	RA (%)
$[M-2H]^{2-}$	1.667,7752	45,5	1667,7791	-2,3	56,7	1667,7778	-1,6	53,7	1667,7762	-0,6	53,8	1667,7749	0,2	50,4	1667,7838	-5,2	51,8
	1.668,2779	94,5	1668,2808	-1,7	100,0	1668,2795	-1,0	97,4	1668,2779	0,0	98,7	1668,2766	0,8	94,9	1668,2855	-4,6	97,2
	1.668,7794	100,0	1668,7822	-1,7	98,7	1668,7809	-0,9	100,0	1668,7793	0,1	100,0	1668,7780	0,8	100,0	1668,7870	-4,6	100,0
	1.669,2809	71,9	1669,2836	-1,6	70,3	1669,2820	-0,7	74,6	1669,2807	0,1	72,7	1669,2792	1,0	76,0	1669,2883	-4,4	73,5
	1.669,7813	39,9	1669,7848	-2,1	40,1	1669,7831	-1,1	44,6	1669,7820	-0,4	42,0	1669,7803	0,6	46,1	1669,7896	-5,0	42,9
	1.670,2813	18,2	1670,2861	-2,9	19,2	1670,2842	-1,7	22,5	1670,2832	-1,1	20,4	1670,2813	0,0	23,6	1670,2909	-5,7	21,0
	1.670,7817	7,9	1670,7873	-3,4	8,1	1670,7852	-2,1	9,9	1670,7844	-1,6	8,6	1670,7824	-0,4	10,5	1670,7921	-6,2	8,9
	1.671,2828	3,0	1671,2885	-3,4	3,0	1671,2862	-2,0	3,9	1671,2856	-1,7	3,2	1671,2834	-0,4	4,1	1671,2933	-6,3	3,4
$[M-2H+4NH_4]^{2+}$	1.703,8470	43,2	1703,8467	0,2	56,2	1703,8455	0,9	52,9	1703,8438	1,9	52,9	1703,8426	2,6	49,6	1703,8514	-2,6	51,0
	1.704,3516	93,3	1704,3484	1,9	100,0	1704,3472	2,6	96,7	1704,3455	3,6	98,0	1704,3442	4,3	94,3	1704,3531	-0,9	96,5
	1.704,8523	100,0	1704,8499	1,4	99,4	1704,8485	2,2	100,0	1704,8469	3,2	100,0	1704,8456	3,9	100,0	1704,8546	-1,3	100,0
	1.705,3536	72,6	1705,3512	1,4	71,3	1705,3497	2,3	75,1	1705,3483	3,1	73,2	1705,3468	4,0	76,5	1705,3559	-1,3	74,0
	1.705,8539	41,5	1705,8524	0,9	40,8	1705,8507	1,9	45,1	1705,8496	2,5	42,5	1705,8479	3,5	46,7	1705,8572	-1,9	43,4
	1.706,3481	26,2	1706,3536	-3,2	19,7	1706,3518	-2,2	22,9	1706,3508	-1,6	20,8	1706,3489	-0,5	24,0	1706,3585	-6,1	21,3
$[M-H+3NH_4]^{2+}$	1.695,3340	53,7	1695,3335	0,3	56,3	1695,3322	1,1	53,1	1695,3305	2,1	53,1	1695,3293	2,8	49,8	1695,3382	-2,5	51,1
	1.695,8385	100,0	1695,8352	1,9	100,0	1695,8339	2,7	96,9	1695,8322	3,7	98,2	1695,8310	4,4	94,4	1695,8398	-0,8	96,7
	1.696,3385	99,6	1696,3366	1,1	99,2	1696,3352	1,9	100,0	1696,3337	2,8	100,0	1696,3323	3,7	100,0	1696,3413	-1,7	100,0
	1.696,8394	80,0	1696,8379	0,9	71,0	1696,8364	1,8	75,0	1696,8350	2,6	73,0	1696,8335	3,5	76,4	1696,8427	-1,9	73,9
	1.697,3401	46,3	1697,3392	0,5	40,6	1697,3375	1,5	45,0	1697,3363	2,2	42,4	1697,3346	3,2	46,5	1697,3440	-2,3	43,3
	1.697,8313	31,9	1697,8404	-5,4	19,6	1697,8385	-4,2	22,8	1697,8375	-3,7	20,7	1697,8357	-2,6	23,9	1697,8452	-8,2	21,2
$[M+2NH_4]^{2+}$	1686,8206	51,7	1686,8202	0,2	56,5	1686,8189	1,0	53,3	1686,8173	2,0	53,3	1686,8160	2,7	50,0	1686,8249	-2,5	51,3
	1687,3229	94,7	1687,3219	0,6	100,0	1687,3206	1,4	97,0	1687,3189	2,4	98,3	1687,3177	3,1	94,6	1687,3266	-2,2	96,9
	1687,8262	100,0	1687,8233	1,7	99,0	1687,8220	2,5	100,0	1687,8204	3,4	100,0	1687,8190	4,3	100,0	1687,8280	-1,1	100,0
	1668,3265	68,8	1668,3247	1,1	70,8	1668,3231	2,0	74,9	1668,3218	2,8	72,9	1668,3202	3,8	76,3	1668,3294	-1,7	73,7
	1688,8272	42,0	1688,8259	0,8	40,5	1688,8242	1,8	44,9	1688,8230	2,5	42,3	1688,8214	3,4	46,4	1688,8307	-2,1	43,1
	1689,3247	25,8	1689,3271	-1,4	19,5	1689,3253	-0,4	22,7	1689,3243	0,2	20,6	1689,3224	1,4	23,8	1689,3319	-4,3	21,1

Table S3. MRM ion transitions of the different CTXs monitored by LC-MS/MS.

Toxin	Precursor ion [M+Na]$^+$ (*m*/*z*)	Product ion [M+Na]$^+$ (*m*/*z*)	Fragmentor (V)	CE (eV)	CAV (eV)
**CTX1B**	1133.6	1133.6	380	40	4
**C-CTX1**	1163.7	1163.7	380	40	4
**2,3-dihydroxyCTX3C**	1079.6	1079.6	380	40	4
**51-hydroxyCTX3C**	1061.6	1061.6	380	40	4
**52-*epi*-54-deoxyCTX1B**	1117.6	1117.6	380	40	4
**54-deoxyCTX1B**	1117.6	1117.6	380	40	4
**49-*epi*-CTX3C**	1045.6	1045.6	380	40	4
**CTX3C**	1045.6	1045.6	380	40	4
**CTX4A**	1083.6	1083.6	380	40	4
**CTX4B**	1083.6	1083.6	380	40	4

Table S4. MRM ion transitions monitored by LC-MS/MS of the different toxic metabolites produced by dinoflagellates as well as accumulated in fish tissue. FP1, FP2 and FP3: Fingerprint ion No.1, No.2 and No.3

Compound	Retention time (min)	ESI	MRM Transitions Q1/Q3 (m/z)		CE (eV)	CAV (eV)
gambierone	8.00	-	$[M-H]^-$/ $[M-H]^-$	1023.5/1023.5	30	5
			$[M-H]^-$/ $[HOSO_3]^-$	1023.5/96.9	60	5
		+	$[M+H]^+$/FP1	1025.5/803.4	33	5
			$[M+H]^+$/FP2	1025.5/219.1	25	5
			$[M+H]^+$/FP3	1025.5/109.1	49	5
MTX4	8.49	-	$[M-2H]^{2-}$/ $[M-2H]^{2-}$	1646.2/1646.2	30	5
			$[M-2H]^{2-}$/$[HOSO_3]^-$	1646.2/96.9	60	5
CTX1B	8.64	+	$[M+NH_4]^+$/$[M+H-H_2O]^+$	1128.6/1093.6	19	5
			$[M+NH_4]^+$/FP1	1128.6/171.2	43	5
			$[M+NH_4]^+$/FP2	1128.6/95.2	55	5
44-methyl gambierone	8.76	-	$[M-H]^-$/ $[M-H]^-$	1037.5/1037.5	30	5
			$[M-H]^-$/ $[HOSO_3]^-$	1037.5/96.9	60	5
		+	$[M+H]^+$/FP1	1039.5/803.4	33	5
			$[M+H]^+$/FP2	1039.5/233.1	25	5
			$[M+H]^+$/FP3	1039.5/109.1	49	5
MTX5	8.87	-	$[M-2H]^{2-}$/ $[M-2H]^{2-}$	1668.8/1668.8	30	5
			$[M-2H]^{2-}$/$[HOSO_3]^-$	1668.8/96.9	60	5
C-CTX1	9.65	+	$[M+H-H_2O]^+$/ $[M+H-2H_2O]^+$	1023.6/1105.6	25	5
			$[M+H-H_2O]^+$/ $[M+H-3H_2O]^+$	1023.6/1087.6	29	5
			$[M+H-H_2O]^+$/ $[M+H-4H_2O]^+$	1023.6/1069.6	37	5
			$[M+H-H_2O]^+$/ FP1	1023.6/191.1	41	5
			$[M+H-H_2O]^+$/ FP2	1023.6/108.9	52	5
gambieric acid C	11.28	-	$[M-H]^-$/$[M-H]^-$	1183.7/1183.7	30	5
		+	$[M+H]^+$/FP1	1185.7/1039.6	17	5
			$[M+H]^+$/FP2	1185.7/943.5	21	5
			$[M+H]^+$/FP3	1185.7/135.1	49	5
gambieric acid D	11.34	-	$[M-H]^-$/$[M-H]^-$	1197.7/1197.7	30	5
		+	$[M+H]^+$/FP1	1199.7/1053.6	17	5
			$[M+H]^+$/FP2	1199.7/957.6	21	5
			$[M+H]^+$/FP3	1199.7/135.1	49	5

C-CTX1-Me	11.40	+	$[M+H-H_2O]^+$/ $[M+H-2H_2O]^+$	1023.6/1105.6	25	5
			$[M+H-H_2O]^+$/ $[M+H-3H_2O]^+$	1023.6/1087.6	29	5
			$[M+H-H_2O]^+$/ $[M+H-4H_2O]^+$	1023.6/1069.6	37	5
			$[M+H-H_2O]^+$/ FP1	1023.6/191.1	41	5
			$[M+H-H_2O]^+$/ FP2	1023.6/108.9	52	5
51-hydroxyCTX3C	12.15	+	$[M+H]^+$/ $[M+H-H_2O]^+$	1039.6/1021.6	15	5
			$[M+H]^+$/FP1	1039.6/155.1	31	5
			$[M+H]^+$/FP2	1039.6/125.1	39	5
52-epi-54-deoxyCTX1B/54-deoxyCTX1B	12.21/12.59	+	$[M+NH_4]^+$/$[M+H-H_2O]^+$	1112.6/1094.6	15	5
			$[M+NH_4]^+$/FP1	1112.6/155.1	39	5
			$[M+NH_4]^+$/FP2	1112.6/125.1	35	5
CTX3C	17.20	+	$[M+H]^+$/ $[M+H-H_2O]^+$	1023.6/1005.6	15	5
			$[M+H]^+$/FP1	1023.6/155.1	27	5
			$[M+H]^+$/FP2	1023.6/125.1	35	5
CTX4A/CTX4B	17.26/17.70	+	$[M+H]^+$/ $[M+H-H_2O]^+$	1061.6/1043.6	15	5
			$[M+H]^+$/FP1	1061.6/155.1	31	5
			$[M+H]^+$/FP2	1061.6/125.1	39	5
